# Evaluation of the distribution of nicotine intravenous injection: an adult autopsy case report with a review of literature

**DOI:** 10.1007/s00414-019-02035-y

**Published:** 2019-04-07

**Authors:** Yayoi Aoki, Tomoya Ikeda, Naoto Tani, Alissa Shida, Shigeki Oritani, Takaki Ishikawa

**Affiliations:** 1 Department of Legal Medicine, Osaka City University Medical School, 1-4-3 Asahi-machi, Abeno, Osaka, 545-8585 Japan; 2 Forensic Autopsy Section, Medico-legal Consultation and Postmortem Investigation Support Center, c/o Department of Legal Medicine, Osaka City University Medical School, 1-4-3 Asahi-machi, Abeno, Osaka, 545-8585 Japan

**Keywords:** Nicotine, Cotinine, Intravenous injection, Acetylcholine receptors, Forensic autopsy

## Abstract

We reported the first comprehensive autopsy case of death due to intravenous injection of nicotine. We examined the distribution of nicotine in the body tissues and fluid and exposed the pathophysiology of nicotine poisoning. A 19-year-old woman was rushed to the hospital in cardiorespiratory arrest and was confirmed dead upon arrival. Liquid nicotine, hydrogen peroxide water, and a syringe were found in the hotel room where she stayed. On autopsy, nicotine concentration was the highest (15,023 μg/mg) in the tissue around the injection mark on the right upper arm. Among the body fluids, the intraperitoneal fluid had the highest, whereas the pericardial fluid had the lowest (0.736 μg/mL) nicotine concentration. Among the organs, the brain had the highest (11.637 μg/mg), whereas the fat tissue had the lowest (1.307 μg/mg) nicotine concentration. The concentration of cotinine, which is the metabolite of nicotine, was the highest in the tissue around the injection mark on the right arm (5.495 μg/mg) and was almost the same among the other body fluids and organs. The respective concentrations of nicotine and cotinine were 1.529 μg/mL and 0.019 μg/mL in the left heart blood and 3.157 μg/mL and 0.002 μg/mL in right heart blood. In this case, the nicotine concentrations in blood reached the lethal level. The distributions of nicotine and cotinine, as indicated by the intravenous injection, were related to the distribution of organs that metabolize nicotine and the distribution of nicotinic acetylcholine receptors.

## Introduction

Nicotine is an amphipathic alkaloid that is contained in familiar products, such as tobacco, nicotine gums, and nicotine patches. It can be highly addictive and is highly toxic [1–5]. Nicotine absorbed into the body or blood vessels migrate to the brain first [6], and this migration is heavily influenced by the distribution of nicotinic acetylcholine receptors [7]. After this migration, nicotine flows via the bloodstream to the other organs, especially the muscles, spleen, liver, and kidneys [8]. Nicotine is metabolized in the liver, and 70% is metabolized to cotinine by enzymes [9]. The half-life of nicotine is approximately 20 min to 2 h, but the half-life of cotinine is 20 h. Moreover, cotinine lodges in the body longer than nicotine [10] before being excreted through the kidneys; notably, 10% of nicotine is excreted in urea without being metabolized [11].

Until now, there had been several reports on cases of poisoning from intraperitoneal nicotine and nicotine-containing products, mainly in infants [12–17]. However, few have reported about poisoning from intravenous nicotine in adults [18, 19]; in fact, there was only one international literature about fatal intravenous nicotine poisoning [20]. In that study, however, a forensic pathologic autopsy was not performed, and there was no information aside from blood concentration (Table 1).

**Table 1:** Review of international literature on cases of nicotine injection, including the present case

Case	Age/Sex	Suicide/murder	Nicotine type	Survival period (h)	Nicotine concentration (blood)	Prognosis	Cause of death/diagnosis	Publication
1	29/M	Suicide	e-Liquid	120	2.1 μg/mL	Death	Nicotine poisoning	Thornton et al. 2014 [20]
2	27/F	Attempted suicide	Cigarette soakage	36	< 0.005 μg/mL	Recovery	Nicotine poisoning	Hagiya et al. 2010 [18]
3	32/M	Attempted suicide	e-Liquid	24	1.3 μg/mL	Recovery	Nicotine poisoning	Sommerfeld et al. 2016 [19]
4	19/F	Unknown	Undiluted nicotine	2	0.002 μg/mL	Death	Nicotine poisoning	Present case

We experienced a rare case of death resulting from intravenous injection of undiluted nicotine. In this case, several body fluids, including blood, and organs were collected. To our best knowledge, this was the first case that reported the distribution of nicotine and cotinine in the body after intravenous nicotine injection.

## Case history

A 19-year-old woman was brought to the hospital in cardiorespiratory arrest. Four and 1/2 h prior, she was noted to be well and about to recuperate in a hotel room where she stayed as a tourist. Two hours and 20 min later, she was found by her husband lying unconscious on the bathroom floor and unresponsive. About an hour after, the emergency response team assessed her to be in cardiorespiratory arrest. She was brought to the hospital but was subsequently declared dead despite appropriate management. At 3 h and 20 min after being proclaimed dead, her rectal temperature was 34 °C at a room temperature of 21 °C.

According to her husband, she did not frequently go to the hospital, although she had symptoms of depression and had experiences of slashing her wrists. She was not a smoker, but she was said to drink alcohol and habitually used to take unknown medicines. A syringe was found on the toilet paper holder in the bathroom. Initially, her husband explained that it was used for mixing some e-Liquid for electronic cigarette. Subsequent police investigation found a green bottle and a green paper case that contained hydrogen peroxide in the room. About 36 h after, a forensic autopsy was performed to investigate the cause of her death.

## Results

### Postmortem imaging

Postmortem computed tomography (CT) showed the presence of moderate to severe pulmonary edema and minimal emphysematous changes in the periphery.

### Autopsy findings

**Fig. 1:**
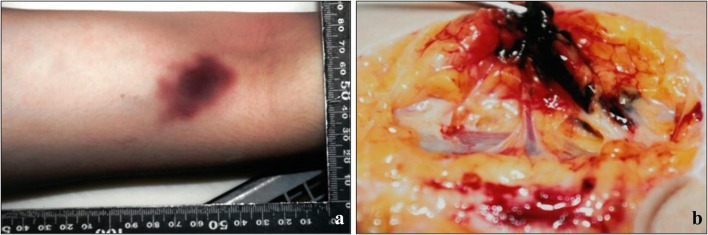
Macropathologic findings in the injection mark on the right upper arm. **a** There is subcutaneous hemorrhage on the right upper arm. **b** The injection marks are along the blood vessel

The subject was 159 cm tall and weighed 45.3 kg, with a body mass index of 17.9. She had dark reddish-purple hypostasis with petechiae on her back. Her face was moderately congested. The palpebral conjunctivae were congested, with few petechiae. Injection marks with subcutaneous hemorrhage were seen on the right upper arm (Fig. 1a) and left elbow fossa and ran along the course of the blood vessel (Fig. 1b). Both left and right heart blood contained thin dark red blood without clots. The brain weighed 1285 g and was edematous and swollen. The left lung weighed 380 g and the right lung weighed 385 g; both lungs were moderately congested and had parenchymal hemorrhage. The other organs were congested. There was no special structural finding.

### Histologic examination

Examination of the lungs showed diffusely edematous and congested intraalveolar spaces and interstitial tissues, some hemorrhages with alveolar injury, and emphysematous changes in the periphery. There were some food debris and bacterial conglomeration from the gastric contents in the bronchioles.

**Fig. 2:**
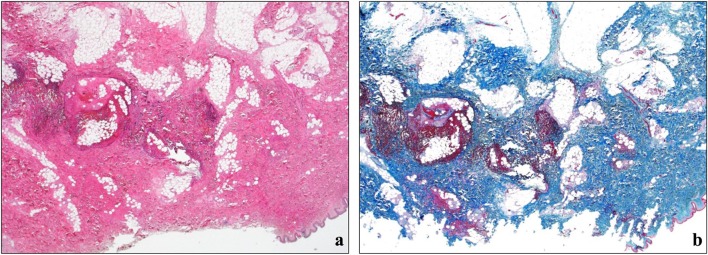
Histopathologic findings of the injection marks on the right upper arm. **a** The mucosal membrane and microvessels in the subcutaneous tissues are damaged and show hemorrhage from the injection (hematoxylin-eosin stain, original magnification × 20). **b** There are no findings of fibrosis and fibroblasts from the old injection marks (azan stain, original magnification × 20)

There were hemorrhages in the subcutaneous tissues around the injection marks. The mucosal membrane and microvessels in the subcutaneous tissues were damaged and showed hemorrhage from the injection (Fig. 2a, b).

### Postmortem biochemistry

The levels of C-reactive protein (0.04 mg/dL) and neopterin (112 pmol/mL), which are both markers of systemic inflammation, were not elevated in the right heart blood [21, 22]. However, the interleukin-6 (IL-6) levels were elevated in the right heart blood (338 pg/mL) and left heart blood (5870 pg/mL). In adult cases, serum IL-6 levels above 1000 pg/mL indicate early-stage systemic inflammation [23, 24]. The concentrations of several serum markers, including creatinine (1.48 mg/dL), blood urea nitrogen (11.6 mg/mL), and uric acid (9.7 mg/mL), were in the forensic pathology normal range. In this case, there was no forensic biochemical evidence of abnormal renal function and skeletal muscle injury, such as rhabdomyolysis [25, 26]. Although the myoglobin concentrations in the cerebrospinal fluid (26,700 ng/mL) and pericardial fluid (662,000 ng/mL) were markedly high, these did not indicate the typical rhabdomyolysis level [27, 28]. The respective pericardial and cerebrospinal fluid catecholamine levels exceeded the pathological cutoff levels (adrenaline, 184,000 pg/mL and 2341 pg/mL; noradrenaline, 205,260 pg/mL and 2741 pg/mL; and dopamine, 8801 pg/mL and 436 pg/mL). The catecholamine levels in the pericardial and cerebrospinal fluids were indicative of drug intoxication [29]. Serum tryptase, which is a marker of anaphylactic shock, was not elevated. The increased levels of immunoglobulin E (95.5 IU/mL), histamine (14.7 ng/mL), and tryptase (1.8 μg/L) were unremarkable [30]. Postmortem biochemical analysis showed no findings indicative of hepatic dysfunction (direct bilirubin, 0.3 mg/dL) and chronic heart failure (human atrial natriuretic polypeptide, 64.4 pg/mL; brain natriuretic peptide, 74.4 pg/mL) [31].

### Toxicologic examination

#### Toxicologic screening

Toxicologic examinations of pericardial fluid samples for drug screening using Triage® (Biosite Diagnostic Inc., San Diego, CA, USA) and gas chromatography–mass spectrometry (GC-MS) were negative.

#### Sample preparation

Samples were extracted using supported liquid extraction (SLE) solid/ liquid phase extraction columns. Standard calibration curves were constructed using blank human serum and 0.1, 0.5, and 1 μg of nicotine and cotinine. Diazepam-D5 (50 μg), which was used as an internal standard, was added to each 0.5 mL sample. Acetonitrile was added in ratios of 0.5 mL to 0.5 mL to blood, 0.3 mL to 0.2 mL to gastric contents, and 0.4 mL to 0.1 mL to bile. Next, the samples were vortexed and centrifuged at 10,000 rpm for 30 s, and the supernatants (250 μL) and aqueous samples (250 μL) were loaded onto the SLE columns. Organs were prepared by tissue homogenization and were centrifuged at 10,000 rpm for 30 s; the supernatants (250 μL) were again loaded onto the SLE columns. After sample loading, the columns were successively washed with 1.3 mL of a mixture of isopropanol and dichloromethane (20:80), followed by 1.3 mL of dichloromethane. The eluates were collected and evaporated to dryness under a gentle stream of nitrogen. The residues were reconstituted in 50 μL of ethyl acetate, and aliquots of the extracts were analyzed by GC-MS using an Agilent model 5975c MSD system equipped with a DB-5MS column (length 30 m; id 0.25 mm; film thickness 0.25 μm; Agilent Technologies, Palo Alto, CA, USA). The analysis conditions were as follows: column temperature, 100 °C to 325 °C; injector temperature, 280 °C; turbo-charged carrier gas, helium at a flow rate of 48 cm/s; and interface temperature, 300 °C. At our facility, the recovery of standards ranged from 60–70% to > 95%. The limit of quantification was defined as the lowest analyte concentration that could be specifically measured and corresponded in the presence of three transitions. Our analyses were based on our previously reported methods [32, 33].

### Results of toxicologic examination

The sample concentrations of nicotine and cotinine quantified by the GC-MS are shown in Tables 2 and 3.

**Table 2:** Distribution of nicotine concentrations in this case

Sample group I (body fluids)		Sample group II (organs)		Sample group III (around the tissue injection mark)	
Sample	Nicotine (μg/mL)	Sample	Nicotine (μg/mg)	Sample	Nicotine (μg/mg)
Intraperitoneal fluid	7.654	Brain (temporal lobe)	11.637	Upper arm (right)	15,023.493
Aqueous humor (left)	6.226	Kidney (right)	5.242	Cubital fossa (left)	1.669
Aqueous humor (right)	5.850	Lung (right lower lobe)	4.808	Forearm (left)	1.040
Pleural effusion (left)	5.654	Ovary (left)	3.984		
Pleural effusion (right)	4.625	Liver	3.704		
Bile	4.446	Spleen	3.615		
Stomach contents	4.269	Heart	3.480		
Heart blood (right)	3.157	Skin (chest)	3.344		
Peripheral blood (iliac vein)	3.109	Uterus	3.229		
Bone marrow	2.701	Pancreas	3.215		
Heart blood (left)	1.529	Ovary (right)	3.130		
Pericardial fluid	0.736	Muscle (pectoral muscle)	2.897		
		Fat tissue (abdominal subcutaneous	1.307

**Table 3:** Distribution of cotinine concentrations in this case

Sample group I (body fluids)		Sample group II (organs)		Sample group III (around the tissue injection mark)	
Sample	Nicotine (μg/mL)	Sample	Nicotine (μg/mg)	Sample	Nicotine (μg/mg)
Stomach contents	0.096	Muscle (pectoral muscle)	0.853	Upper arm (right)	5.495
Aqueous humor (right)	0.096	Lung (right lower lobe)	0.353	Forearm (left)	0.133
Pleural effusion (left)	0.048	Liver	0.280	Cubital fossa (left)	0.114
Pleural effusion (right)	0.043	Uterus	0.200		
Pericardial fluid	0.035	Ovary (right)	0.181		
Bile	0.034	Pancreas	0.178		
Aqueous humor (left)	0.026	Spleen	0.138		
Heart blood (left)	0.019	Ovary (left)	0.135		
Peripheral blood (iliac vein)	0.015	Heart	0.132		
Bone marrow	0.014	Brain (temporal lobe)	0.125		
Intraperitoneal fluid	0.003	Skin (chest)	0.104		
Heart blood (right)	0.002	Fat tissue (abdominal subcutaneous fat)	0.054		
		Kidney (right)	0.028		

#### Body fluids

Nicotine: The concentration was the highest in the intraperitoneal fluid (7.65 μg/mL) and the lowest in the pericardial fluid (0.73 μg/mL). The concentration in the left heart blood (1.52 μg/mL) was lower than those in the right heart blood (3.15 μg/mL) and the iliac vein blood (3.10 μg/mL) (Table 2; sample group I).

Cotinine: All samples showed low concentrations of less than 0.1 μg/mL. There was no significant difference among the samples (Table 3; sample group I).

Ethanol: No significant concentrations of ethanol and other drugs were detected in both the left heart and right heart blood, as well as in the gastric contents.

#### Organs

Nicotine: The highest concentration was in the temporal lobe of the cerebrum (11.63 μg/mg), followed by the right kidney (5.24 μg/mg) and the lung (4.80 μg/mg). There was no significant difference in the nicotine concentrations among the other organs (2.89–5.24 μg/mg). The least concentration was in the fat tissues (1.30 μg/mg) (Table 2; sample group II).

Cotinine: The concentration was the highest in the greater pectoral muscle (0.85 μg/mg) and the lowest in the right kidney (0.028 μg/mg). There was no significant difference in the nicotine concentrations among the other samples (0.05–0.35 μg/mg) (Table 3; sample group II).

#### Tissues around the injection marks

Nicotine: The concentration in the tissue around the injection site on the right upper arm (15,023 μg/mg) was the highest. The concentrations in the other injection sites were 1.66 μg/mg in the left cubital fossa and 1.04 μg/mg in the left forearm (Table 2; sample group III).

Cotinine: The concentration was the highest in the tissue around the injection site of nicotine on the right upper arm (5.49 μg/mg). However, the concentrations in the other tissues around the injection site were not significant (left cubital fossa, 0.11 μg/mg; left forearm, 0.13 μg/mg) (Table 3; sample group III).

#### Hydrogen peroxide solution

The left and right heart blood and aqueous humor concentrations of the hydrogen peroxide solution found in the room were quantified using hydrogen peroxide colorimetric detection kit (Cosmo Bio Co., Ltd., Tokyo, Japan) and Multiskan FC (Thermo Fisher Scientific, MA, USA) [34, 35]. The concentrations in the left (31.43 μM) and right (46.82 μM) heart blood and iliac vein (27.78 μM) were within the normal range of a healthy person. Furthermore, the concentrations of hydrogen peroxide were 10.27 μM in the pericardial fluid; 10.04 μM in the left and 10.29 μM in the right pleural fluid; and 5.39 μM in the left and 5.53 μM in the right vitreous humor.

## Discussion

In the present case, there were injection marks on the bilateral upper limbs. Notably, the nicotine concentration was extremely high at 15,023 μg/mg in the tissues around the injection mark on the right upper arm and reached a lethal level of 1.52 to 3.15 μg/mL in the blood (lethal cutoff > 1.4 μg/mL) [36–38]. The lungs were edematous and congested, with alveolar parenchyma hemorrhage, and the brain was swollen. Therefore, we concluded that the cause of death was intravenous nicotine poisoning. The body fluid drug screening test by GC-MS revealed the presence of nicotine and its metabolite cotinine, but no other drugs were detected. However, we could not show scientific evidence of suicide.

The green bottle found in the room was confirmed to be hydrogen peroxide, although its level (5.39–46.82 μM) was within the normal range (13–57 μM) [39, 40]. Therefore, the hydrogen peroxide probably did not contribute to her death. Moreover, the markers of anaphylactic shock were not increased and there was no scientific evidence of rhabdomyolysis, based on the myoglobin biochemical examination and immunohistochemistry of the skeletal muscle [41, 42].

Several case reports were on oral ingestion of nicotine [43, 44]. However, in the present case the gastric concentration of nicotine was low. Moreover, the levels of nicotine and cotinine were higher in the left than in the right heart blood. The levels of cotinine, which has a long half-life, were low in almost all the samples, but the nicotine level in the injection mark on the right upper arm was extremely high. These findings implied that the nicotine was taken intravenously in the present case.

In general, the blood concentration of nicotine reaches approximately 10 ng/mL within a few minutes of smoking one cigarette; in the present case, the nicotine concentration in the blood samples was above that level. Intravenous injection of nicotine likely caused the death in the present case. The moment nicotine enters the body, it is metabolized into cotinine, which reaches a concentration that is 2–4 times higher than that of nicotine within an hour. In the present case, the higher levels of nicotine than cotinine in all samples suggested an acute death due to the high concentration injected nicotine.

In the present case, the nicotine concentrations varied among the samples. Nicotine that enters the body through the bloodstream is immediately distributed to the brain and is metabolized for excretion in the kidney. The resulting distribution of nicotine is affected by the acetylcholine receptors in the body. The nicotinic acetylcholine receptor, which is an ionotropic receptor, is widely distributed in the brain; through its ligand, nicotine binds to the receptors to make changes in the body [24, 45]. These receptors are also distributed in the nerves. Nicotine is also distributed in other sites, such as fat tissue [46], where it controls the production of adiponectin. In the present case, the lower nicotine level in the fat tissue than in the brain suggested that nicotine migrated to the brain within a few minutes after injection. The affinity of nicotine is higher to acetylcholine receptors in the brain than to fat tissue. However, nicotine is mainly metabolized in the liver not in the brain; therefore, the level of cotinine is expected to be low in the brain. In this case, there was no significant difference in the nicotine concentrations among the organs related to metabolism. This can probably be explained by the fact that the time it takes to metabolize nicotine depends on the injection speed and amount [29].

Recently, the methods of nicotine intake had been changing with the increasing use of electronic cigarettes. This means that some products that contain high concentrations of nicotine can be easily acquired, and nicotine-related fatality rates can increase. A large database on the body distribution of nicotine and cotinine, based on quantification from as many samples as possible, will help estimate the route of nicotine intake in the field of forensic medicine and can be clinically useful for the medical treatment of acute nicotine poisoning cases.

## Data Availability

All data used to support the findings of this study are included within the article.
